# Evaluating the relationship between adult attention-deficit/hyperactivity disorder and riding behavior of motorcyclists

**DOI:** 10.5249/jivr.v11i1.1098

**Published:** 2019-01

**Authors:** Homayoun Sadeghi-Bazargani, Kamal Hasanzadeh, Shaker Salarilak, Shahrokh Amiri, Mina Golestani, Nasrin Shahedifar

**Affiliations:** ^*a*^Department of Statistics and Epidemiology, Tabriz University of Medical Sciences, Tabriz, Iran.; ^*b*^Department of Public Health, Islamic Azad University (Tabriz Branch(, Tabriz, Iran.; ^*c*^Department of Psychiatry, Tabriz University of Medical Sciences, Tabriz, Iran.; ^*d*^Road Traffic Injury Research Center, Tabriz University of Medical Sciences, Tabriz, Iran.; ^*e*^Tabriz International Safe Community Support Center, Tabriz, Iran.

**Keywords:** Road safety, Adult ADHD, Motorcyclists’ riding behavior, Attention Deficit, Hyperactivity, Disorder

## Abstract

**Background:**

Motorcycling is one of the main causes of injury, and motorcyclists are vulnerable to road traffic injuries. Attention Deficit Hyperactivity Disorder (ADHD) in adults is presumably one of the determinants of road traffic injuries and motorcyclists’ risky behavior. Despite the few studies on the relationship between motorcycle injuries and adult ADHD, their association has not been investigated using standardized instruments. This study aimed to analyze the relationship between motorcyclists’ adult ADHD and risky riding behaviors.

**Methods:**

This community-based, cross-sectional study was performed on 340 motorcyclists in Bukan city, west Azerbaijan province, Iran in 2015 and 2016 using a cluster-random sampling in seven areas of the city. According to the city map used by Bukan’s Health Centers, the city was divided into 14 clusters. Then, seven clusters (out of 14) were selected randomly. To reach the anticipated sample size, the data were collected from these seven clusters. In this study, the data collection instruments were: standard Motorcycle Rider Behavior Questionnaire (MRBQ), Conners' Adult ADHD Rating Scales (CAARS) questionnaire and a checklist designed by the researchers. The Stata 13 software package was used to analyze the collected data. Pearson correlation coefficient and multiple linear regression were performed to study the linear relationship between ADHD screening and MRBQ scores.

**Results:**

All 340 participants were male and the mean age was 30.2 years (SD=9.1). In addition, 22.1% of motorcyclists had a history of motorcycle crash. Bivariate analysis showed a significant association between risky riding behaviors and age, motorcycling records, and mean of riding hours per day (P-value less than 0.05). Multivariate analysis confirmed the correlation between ADHD and risky riding behaviors in all subscales (A, B, D) (p less than 0.05).

**Conclusions:**

Those with a high ADHD screening score are more likely to have risky riding behaviors.

## Introduction

Traffic crashes represent a major challenge to public health; ^1^ they cause serious injuries to 20-50 million people and the death of 1.24 million around the world annually. It is predicted that the annual death toll of injuries will rise to 1.9 million in 2030 if no effective actions and measurements are taken.^2^ Though death and injury rates have declined in developed countries over the last few years, low- and middle-income countries (LMICs) have simultaneously shown an increase.^3-5^ Nevertheless, few studies have been conducted on this issue and they do not provide enough strong evidence to improve road traffic safety in all related aspects, in developing countries. ^2,3,6^ It has therefore been concluded that this problem has not been taken into consideration properly.^2,3^ About 62% of deaths caused by road traffic injuries are reported from 10 countries including Iran.^7^ Like other Asian countries, motorcyclists in Iran are among the most vulnerable groups.^8^ Compared to other drivers, they are at a higher risk of injuries; for example 7 times more than car drivers and 5.5 times more than truck drivers.^9^ Undoubtedly, to reduce injuries and death tolls caused by injuries in LMICs including Iran, motorcyclists should be given appropriate priority.^10,11^ Despite their high vulnerability in Iran,^9^ there is not enough applied research in related fields.^12^ Road traffic injuries are a major but neglected public health challenge requiring efforts for sustainable prevention. The road traffic system is the most complex and the most dangerous system that people have to negotiate.^13^

With a systemic glance ^14^ at motorcycle accidents, three main components can be ascertained, including: factors related to vehicles (such as motorcycles), environmental factors (such as traffic conditions, road types and conditions, weather situation), and human factors (such as riders).

Although there is plenty of research on road safety resulting in many findings on the first two factors, few studies have been conducted on the third factor addressing how human factors, especially psychological factors, can lead to risky riding behaviors and motorcycle injuries.^15^ Attention Deficit Hyperactivity Disorder (ADHD), one of the most common psychiatric disorders in childhood and adolescence,^16^ is a serious, chronic and debilitating mental disorder. It affects about 2.8 to 3.9 million school children in the US and may continue into adulthood.^17^ About 50% to 65% of children with ADHD show symptoms of the disease in their adulthood.^18^ Its prevalence in adults is four percent.^19^ Adults with ADHD may be distracted by the slightest stimuli, take impulsive decisions in the case of riding behaviors, and be prone to multiple and severe injuries.^20^ Barkley et al. indicate that riders with adult ADHD show a high level of inattention in riding test.^21^ Other studies reveal that motorcyclists with adult ADHD display abnormal behavior such as speeding, riding without license and causing crashes.^22-24^ Since studies on the relationship between motorcyclists’ adult ADHD and risky riding behavior using standard tools are rarely available, this study aims to determine the association between adult ADHD score and risky motorcycle riding behavior.

## Methods 

***Study type and participants***

The present cross-sectional survey was conducted on 340 motorcyclists in Bukan city, west Azerbaijan province, Iran, from January 2015 to January 2016. By means of random cluster sampling, the city was divided into 14 clusters according to the geographical areas covered by urban health centers; then seven clusters were selected randomly. The data were collected from motorcycle repair shops, motorcyclists’ homes and workplaces in each cluster until the expected sample size was obtained. The sample size of 340 participants was gathered equally from the seven selected clusters. Sample size was estimated using Sampsi command of Stata V.11. Considering the most similar available study conducted by Abedi et al., assuming a standard deviation of 22.96, 95% confidence level and an accuracy of^3,12^ the primary sample size of 227 was estimated. Finally, it was multiplied by a design effect coefficient of 1.5. Then the final sample size of 340 was calculated.

***Inclusion and exclusion criteria***

The inclusion criteria in the study were as follows: 

1. The interviewee rode a motorcycle at least 3 times a month. 

2. The interviewee was over 15 years old.

3. The interviewee was a resident of Bukan City.

4. The interviewee was alert at the time of completing the questionnaire. 

The exclusion criteria were as follows:

1. Having no motorcycle riding experience in the last month.

2. Having a medical history of major mental disorders in the past.

3. Lacking informed consent to participate in the study. 

4. Lacking motivation to participate in the study and complete the questionnaire. 

***Tools***

The main variables, study outcomes and measuring tools are explained as follows:

1. Background variables included age, sex, marital status, education level, and socio-economic status. The available Persian tool of SESIran (ultrashort version) was used in order to measure socio-economic status (SES).^25-27^

2. Variables related to motorcycle riders included having a motorcycle license, the average riding hours a day, the average number of motorcycle riding days a week.

3. Riding behavior assessment variables included riding on the wrong side of the road, speeding, not wearing a helmet, carrying unauthorized cargo, and other dangerous riding behaviors such as tailgating and doing acrobatics. The standard questionnaire of MRBQ was used to assess the behavior. Both validity and reliability of the questionnaire had already been evaluated by Elliott et al. in 2007,^15^ and translated and adapted by Motavallian et al. in Persian in 2009^28^ The questionnaire consists of 48 items and the scores ranges between 0 and 192. The answer options are based on 5-point Likert scale: (never=0), (rarely=1), (sometimes=2), (often=3), and (most of the time=4). Behavior scores of the questionnaire were normalized into a range of 0 to 100. The Cronbach’s alpha for subgroups was estimated to be in a range of 73-93%.

4. The short-form Persian questionnaire of Conners' Adult ADHD Rating Scales (CAARS) was used to measure ADHD variables. This questionnaire includes 30 questions and four subscales including attention disorder (subscale A), impulsivity index (subscale B), general index of ADHD symptoms and lack of attention (subscale C), and ADHD index (subscale D). This scale was translated into Persian in Tabriz in 2013.^29^ The answer options were based on 4-point Likert scale including (0: almost never, never), (1: occasionally, sometimes), (2: most of the times, usually), and (3: very often, always). The internal consistency of the Persian version was 82-97% based on Cronbach’s alpha for its subscales. The content validity was confirmed for all four subscales using modified Kappa over 0.76.

***Statistical analysis***

The data were analyzed using the Stata v.13 statistical software package (Texas Stata Corp.) and using descriptive statistical methods such as reporting frequencies, percentages, means and standard deviations. The point estimates and their 95% confidence intervals were calculated and reported for key variables. Through a preliminary bivariate analysis, the relationship between ADHD screening score and riding behavior score was assessed using Pearson correlation coefficient. Three complementary multivariate statistical models were developed including various ADHD subscales in each model. Multiple linear regression analysis was done using normalized scores of MRBQ as the outcome variable. Other potential confounders and cofactors were included in constructing the multivariate models (if p-value <1). The score extracted from subscale D of Conner’s questionnaire was used as the main predictor variable (model 1) and subscales A and B were considered as the main predictors in the other two models. The three subscales were not used simultaneously in a single model due to their intrinsic mutual multicollinearity. 

Additionally, in order to evaluate the multivariate analysis models, the normal distribution of errors was examined by obtaining and plotting residuals; the multicollinearity between variables was examined by estimating a variance inflation factor (VIF); and the linear relationship between independent and dependent variables was tested by drawing scatter plots. All the statistical tests were interpreted as two-sided, with statistical significance level below 0.05. 

## Results

In the current study, 340 motorcyclists were examined. All participants were male (mean age: 30.2 years; 95% CI: 29.2-31.2); and 32% of them had an academic education (95% CI: 27.8-37.8). Regarding a history of riding motorcycles, 9.7% of participants reported riding experience for less than 6 months (95% CI: 6.7-13.4), 10.6% reported 6-12 months (95% CI: 7.7-14.4) and those with more than 12 months riding experience comprised the remainder 79.7% (95% CI: 75%-84%). 67.3% of participants reported riding a motorcycle more than 4 days a week, and 15.6% reported consistent use of a helmet. The average hours of riding was 2.1 per day and the maximum MRBQ score was 116. The most common risky behaviors by most of the survey participants were as follows:

1. Poor control of the motorcycle in turns (for example U-turns)

2. Riding with lights off in the dark

3. High speeding on freeways

4. Running a red light

5. Carrying heavy loads

**Figure 1:**
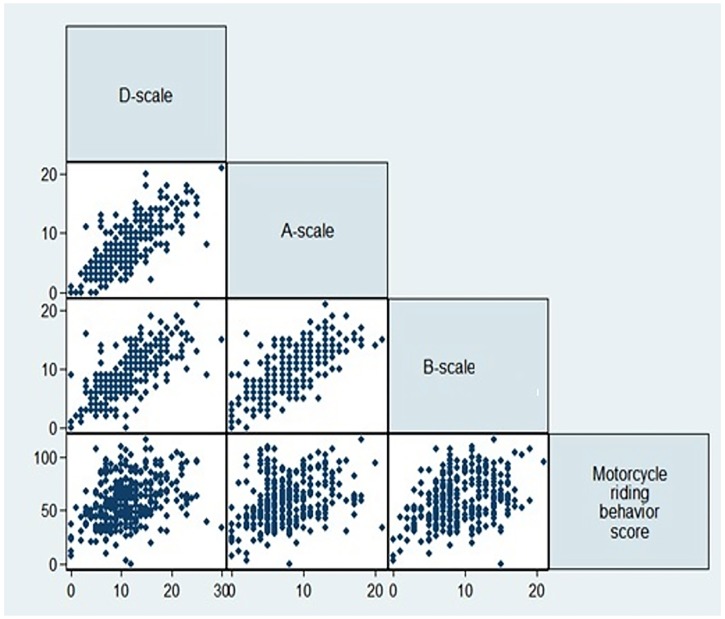
Multiple Scatterplots of Association among Motorcycle Riding Behavior and ADHD Subscale.

A linear correlation pattern was observed between riding behaviors of motorcyclists and adult ADHD scores (Figure 1). Using Pearson correlation coefficient, a positive and significant correlation was observed between motorcyclists’ riding behaviors and adult ADHD scores in all independent subscales (A, B, D), (P-value <0.05) (r=0.4). The maximum correlation belonged to the subscale B in the age group 30-45 years (r=0.8), and the minimum correlation belonged to the subscale A in the age group 17-30 years (r=0.3). In determining the relationship between ADHD and motorcyclists’ riding behavior, bivariate analysis showed a significant association with age, records of riding a motorcycle, and average riding hours per day. The results of multivariate analysis confirmed the relationship between adult ADHD and motorcyclists’ riding behavior in all subscales (A, B, D). Bivariate and multivariate analyses are shown in Table 1,  2 and 3.

**Table 1:** Correlation coefficients between motorcycle riding behavior score and subscales of ADHD.

Hyperactivity Score	Pearson Correlation Coefficient between Riding Behavior Score and ADHD		Spearman Correlation Coefficient between Riding Behavior Score and ADHD	
	Correlation Coefficient	P-value	Correlation Coefficients	P-value
Subscale A Score	.32	.001	.33	.001
Subscale B Score	.78	.001	.80	.001
Subscale C Score	.37	.001	.38	.001
Subscale D Score	.36	.001	.39	.001
Total Score of AD	.38	.001	.40	.001

**Table 2:** Univariate analysis of predictive factors of motorcycle riding behavior.

Variables	Behavior Score	
	Regression Coefficient	P-value
Age	-.45	.001
subscale A, ADHD	1.68	.001
subscale B, ADHD	2.02	.001
subscale D, ADHD	1.42	.001
**Average hours of riding per day**		
2-4 hours	10.34	.009
4-6 hours	21.05	.001
6-8 hours	19.45	.002
More than 8 hours	-25.25	.23
**Motor riding record**		
Less than 6 months	18.94	.001
6-12 months	13.53	.001
More than 12 months	.16	.001

**Table 3:** Multivariate analysis of predictive factors of motorcycle riding behavior.

Variables		Behavior score		
		Regression Coefficient	Standard Beta	P-value
**Model I: **				
**ADHD Index (Subscale D) as the main predictor of motorcycle riding behavior**				
	subscale D, ADHD	1.09	.28	.001
**Average hours of riding per day**				
	2-4 hours	9.8	.18	.008
	4-6 hours	14.4	.16	.008
	6-8 hours	15.9	.16	.007
	More than 8 hours	-12.6	-.03	.520
**Motorcycle riding record**				
	Less than 6 months	11.20	.15	.004
	6-12 months	10.34	.14	.003
	More than 12 months	12.3	.17	.002
	Age	-.20	.08-	.08
**Model II: **				
**Attention Deficit Index (Subscale A) as the main predictor of motorcycle riding behavior**				
	subscale A, ADHD	1.34	.26	.001
**Average hours of riding per day**				
	2-4 hours	10.12	.19	006
	4-6 hours	.16	.18	.003
	6-8 hours	15.9	.16	.007
	More than 8 hours	-14.44	-.036	.46
**Motorcycle riding record**				
	Less than 6 months	12.38	17	001
	6-12 months	11.27	.16	.001
	More than 12 months	14.32	.13	.004
	Age	-.24	-.10	.03
**Model III: **				
**Hyperactivity-Impulsivity Index (Subscale B) as the main predictor of motorcycle riding behavior**				
	Subscale B, ADHD	1.75	.33	.001
**Average riding hours per day**				
	2-4 hours	10.34	.19	.004
	4-6 hours	15.40	.18	.004
	6-8 hours	18.65	.18	.001
	More than 8 hours	-8.02	-.02	.67
**Motorcycle riding record**				
	Less than 6 months	11.95	.16	.002
	6-12 months	11.48	.16	.001
	More than 12 months	15.2	.14	.003
	Age	.15	-.06	.18

## Discussion

According to the findings, the higher ADHD screening scores, the riskier the motorcyclists’ behavior. Some problems such as distraction, lack of focus, intolerance for waiting, nervousness, irritability and risk-taking may affect motorcyclists’ riding behaviors.^20^ A couple of studies^30,31^ clarify that risky riding behaviors decline after the disorder is cured, confirming the relationship between ADHD and risky behaviors. Furthermore, all types of crashes and injuries in patients with ADHD were more frequent compared to the control group.^32^ However, a clinical trial study discovered an independent relationship between adult ADHD and risky behaviors when riding.^33^

The multivariate analysis and multiple scatter plots revealed the maximum correlation between B subscale score (with symptoms of impulsivity and hyperactivity) and risky riding behaviors (r=0.80), and the minimum correlation between D subscale score (with symptoms of impulsivity and hyperactivity) and risky behaviors. The possible reason could be that MRBQ is most often used to measure impulsivity and active riding behavior of motorcyclists. Also, motorcyclists with ADHD are more prone to impulsive and sudden behaviors such as doing a wheelie and riding at high speeds. The behavior scores related to A and D subscales increased and reached their peaks at the age of 45 years. Such pattern would not be observed among riders older than 45 years since the pattern of this disorder changes with aging. In other words, as people get older, impulsivity and hyperactivity behaviors are replaced by behaviors like laziness, restlessness and disquiet.^34^ Accordingly, hyperactive teenagers experience more traffic crashes than other teenagers,^35^ as well as hyperactive adolescents compared to others.^36,37^

Comparing to another study of 46 motorcyclists involved in crashes indicated no significant relationship between ADHD and motorcycle crashes.^38^ Conversely, our study could highlight different age ranges as factors affecting both different crash-cause behaviors as well as ADHD subscales.

The findings of a study conducted by Safiri et al. (2011) were in line with the results of the current study because both studies indicated that adults with ADHD behaved in more risky ways which could lead to crashes causing trauma.^39^

A study conducted in 2011 found that there is an inverse relationship between motorcyclists’ ADHD screening scores and the rate of helmet use.^40^ This is consistent with our finding that the greater the ADHD screening score, the lower the frequency of helmet use.

Contrary to the results of the current study, subscale A with attention deficit symptoms played a preventive role in injuries caused by motorcycle crashes in another study. This could be influenced by participants’ economic situations as well as the diversity of motives and purposes to use motorcycles.^41^

According to the current study, motorcyclists with ADHD experienced more risky behaviors than others. Moreover, hyperactivity can be considered as a risk factor too. So, screening and diagnosis of this psychological disorder in vehicle drivers, especially motorcyclists, would play a significant role in reducing risky behavior patterns and subsequent crashes and injuries.

In this study, identification of personality disorders that could affect risky riding behaviors was not possible due to the lack of psychiatric interview and screening. This could be the main limitation of the current study.

**Acknowledgements**

The authors appreciate the friendly and sincere participation and collaboration of Dr. Leili Abedi, Dr. Mahmoodi and all other colleagues who helped us to conduct this research. 
